# Some Features of Exophthalmic Goitre

**Published:** 1909-03-06

**Authors:** T. D. Savill

**Affiliations:** Physician to the West End Hospital for Nervous Diseases


### Hospital Clinics.

### SOME FEATURES OF EXOPHTHALMIC GOITRE.

By T. D. SAVILL, M.D.Lond.; Physician to the West End Hospital for Nervous Diseases.

(Notes of a Clinical Lecture at the West End Hospital, Welbeck Street.)

There have lately been several very good examples of exophthalmic goitre attending the hospital, and I have got several of them together to show to-day. The first is a good instance of what I may call classical Graves' disease. Very briefly, her history is, that eighteen months ago she first suffered from anæmia and amenorrhœa, and twelve months ago she first noticed that her eyes were bulging. She is now 21 years of age, and three years ago she says she had pain after food, and was told she had gastric ulcer.

She now presents symptoms of all four of the kinds into which the manifestations of this disease can be divided. That is, she has, first, an enlargement of the thyroid. This enlargement is uniform and equal on the two sides, as it commonly, but not always, is in these cases. The thyroid has diminished in size since she came into the hospital five weeks ago, although by measurement the diminution of the size of the neck is only half an inch. But a better guide than measurement is the patient's own sensations, and the softer consistence of the mass when it is palpated.

Next, she has proptosis. What this symptom is due to is still but vaguely understood. It is generally ascribed to vaso-motor dilatation of the vessels at the back of the orbit; and there is also a considerable increase in the amount of fat in that situation. Connected with the eyes in Graves' disease there are various signs, about half a dozen, to which the names of certain observers have been attached. There is, for instance, von Græfe's sign, which is the appearance of an intervening band of white sclerotic between the lid and the upper margin of the iris when the eyes are moved downwards. All these signs are really quite insignificant; for they all amount to this, that the eye is pushed forward, as anyone can see for himself, and so it has difficulty in performing the normal movements.

The third of the four cardinal symptom groups is cardio-vascular irritability. Her pulse rate has averaged 100 to 120 per minute since she has been in the hospital, and is at the present moment much more, probably almost 200. Besides tachycardia she suffers from palpitation; but she has no valvular disease, which is as a rule absent in these cases except as a complication. Fourthly, there are the symptoms of nervous excitability, which is more marked in some patients than in others, and is most frequently evidenced by tremor, sleeplessness, and nervousness. It is on account of these nerve symptoms that we get so many cases of exophthalmic goitre at this hospital. In this particular patient the nervous system betrays little beyond tremor, and even inability to sleep is absent. Trophic skin lesions, which are not common in this disease, are also absent. At the present time the patient is unable to walk, and this she puts down to her five weeks' rest in bed; she could walk on admission to hospital.

The next patient is one who has been under treatment longer. She is 24 years of age, and came originally complaining of "nervousness." This symptom is really more constant than any of the others, and a large proportion of the patients come complaining of it. Next to nervousness in point of frequency come the cardio-vascular symptoms, with thyroid enlargement next after them, and proptosis last of all. This patient, when she was first seen, had thyroid enlargement, though she has none now. She had no proptosis, but a symptom which puzzled us very much at first, namely nystagmus. This turned out to be congenital and not connected in any way with her disease. She had also at one time tachycardia, and at the present instant her pulse rate is 120; but it is often now not much more than 70, and has probably gone up for the moment under the influence of excitement.

The diagnosis of exophthalmic goitre is generally pretty simple. It is only likely to be mistaken for other diseases which produce a moderately fine tremor, such, for instance, as neurasthenia. It was, in fact, the resemblance between these cases of thyroid enlargement—exophthalmic goitre, and those of neurasthenia which first gave me the notion some twelve or fourteen years ago that neurasthenia and toxæmia are causally related.* There are various other conditions which have to be thought of in making the differential diagnosis, and among them are disseminated sclerosis, alcoholic tremors, and various hysterical conditions associated with tremor. When I first saw this particular girl I was not sure whether the case was one of disseminated sclerosis, hysteria, or Graves' disease.

This case is especially instructive from the point of view of treatment. Now the treatment of exophthalmic goitre is a somewhat large subject, and many remedies have been tried and advocated. When that is the case it is generally a sign that nothing does any good, but it is not so in this disease. There are quite a number of patients with Graves' disease who are benefited by some drug or another. This girl is unfortunate in that she has *not* received much benefit from any of these drugs. She has had in turn Kocher's treatment by phosphate of soda, 15 grains three times a day; aspirin; salicylate of bismuth; adrenalin; calcium chloride, 20 grains twice a day; calcium lactate; arsenic; ergot; radogen (which is the dried milk of thyroidectomised goats); and thyroidectin (which is the serum of goats similarly treated). She has also had the constant current applied to her neck. It is true that she has improved, but only as much as one expects any patient to improve under the rest and regular life of a

* "Lectures on Neurasthenia." Fourth edition. 1908. H. J. Glaisher and Co., London.

hospital ward. I cannot honestly say that any one of these drugs has done her any good.

I am now going to try the effect of knocking off all meat and milk, and I will explain why I propose to do this. The disturbance of both the nervous and cardio-vascular systems is a generalised one, and we suspect, therefore, that some toxæmic blood condition is at the bottom of it. Moreover, when our attention is directed to the thyroid we remember that this is a gland with a most important internal secretion, and that its diminished activity leads to a disease, myxœdema, which corresponds in a very close manner with the other condition of hyper-thyroidism, or Graves' disease. For the effects of the thyroid secretion upon the nervous system, the cardio-vascular system, the skin, and the hair are exactly opposite in the two diseases. In short, both are due to something that is wrong with the internal secretion of the thyroid. Professor Starling and other physiologists have shown that the thyroid secretes a product known as iodothyrin, which belongs to the group of bodies called hormones, and it has an effect on the tissues I have named. If, therefore, removal of the thyroid produces a condition the opposite of that caused by iodothyrin, when there is reason to suppose that the latter is present to excess, the patient's diet should be deprived of all animal products; that is, chiefly milk and meat. We shall also, in addition to this *régime*, give a further trial to the constant current.

Here is another patient; and now we pass from a classical case to one which might be called *pseudo*-Graves' disease. This patient is 24, and came for shortness of breath, tremor of both hands, and tachycardia; according to the notes she had von Græfe's sign. Her pulse rate was 140, and she had marked swelling of the thyroid. This patient has had very bad teeth for a long time, and one finds by experience that very severe pyorrhœa alveolaris occurs in a certain proportion of sufferers from the disease. Further, the pyorrhœa has preceded the development of the symptoms of Graves' disease, and the relief of pyorrhœa has been attended by disappearance of the thyroid symptoms. These cases are difficult to differentiate from those of true Graves' disease, from which they differ but little, and chiefly then by the predominance of nervous symptoms, which may amount even to absolute mania. I prefer to call these cases pyorrhœal exophthalmic goitre. In this patient the pyorrhœa was not so marked as in some that one sees.

The next patient is a woman of 40 who had symptoms of Graves' disease more than ten years ago, and who has come up quite by chance to-day. There was proptosis and swelling of the neck then, and the condition of the teeth was exceedingly bad. The nervous symptoms were also very prominent, and she was seldom able to attend the hospital alone. If she went out unaccompanied she was subject to agoraphobia, and would run home in a panic. The mental symptoms also were severe. Twice she had to go into an asylum for several months owing to her uncontrollable and irresponsible mental state. She still exhibits tremor, but has now no thyroid enlargement. Before the goitrous symptoms began she suffered from severe pyorrhœa, and the goitre disappeared after this was cured. She illustrates well the fact that some of these patients with profound mental disturbance are capable of cure. It was a long lane, but we found a turning in the end. She has, in fact, improved on treatment directed towards gastro-intestinal antisepsis and towards removal of her pyorrhœa alveolaris.

